# Multiple modulatory roles of serotonin in chronic pain and injury-related anxiety

**DOI:** 10.3389/fnsyn.2023.1122381

**Published:** 2023-04-18

**Authors:** Shun Hao, Wantong Shi, Weiqi Liu, Qi-Yu Chen, Min Zhuo

**Affiliations:** ^1^Department of Pharmacology, School of Pharmacy, Qingdao University, Qingdao, Shandong, China; ^2^Oujiang Laboratory, Zhejiang Lab for Regenerative Medicine, Vision and Brain Health, Wenzhou, Zhejiang, China; ^3^International Institute of Brain Research, Forevercheer Medicine Pharmac Inc., Qingdao, Shandong, China; ^4^Center for Neuron and Disease, Frontier Institute of Science and Technology, Xi’an Jiaotong University, Xi’an, Shaanxi, China; ^5^The Brain Cognition and Brain Disease Institute, Shenzhen Institute of Advanced Technology, Chinese Academy of Sciences, Shenzhen, Guangdong, China; ^6^Department of Physiology, Faculty of Medicine, University of Toronto, Toronto, ON, Canada

**Keywords:** 5-HT, 5-HT receptors, chronic pain, anxiety, synaptic modulation, ACC, insular cortex, amygdala

## Abstract

Chronic pain is long-lasting pain that often persists during chronic diseases or after recovery from disease or injury. It often causes serious side effects, such as insomnia, anxiety, or depression which negatively impacts the patient’s overall quality of life. Serotonin (5-HT) in the central nervous system (CNS) has been recognized as an important neurotransmitter and neuromodulator which regulates various physiological functions, such as pain sensation, cognition, and emotions–especially anxiety and depression. Its widespread and diverse receptors underlie the functional complexity of 5-HT in the CNS. Recent studies found that both chronic pain and anxiety are associated with synaptic plasticity in the anterior cingulate cortex (ACC), the insular cortex (IC), and the spinal cord. 5-HT exerts multiple modulations of synaptic transmission and plasticity in the ACC and the spinal cord, including activation, inhibition, and biphasic actions. In this review, we will discuss the multiple actions of the 5-HT system in both chronic pain and injury-related anxiety, and the synaptic mechanisms behind them. It is likely that the specific 5-HT receptors would be new promising therapeutic targets for the effective treatment of chronic pain and injury-related anxiety in the future.

## Introduction

Chronic pain is defined as any pain that lasts for several weeks, or longer, and persists even after the pathogenic elements have receded. It is often categorized as neuropathic pain, which is caused by nerve damage, and nociceptive pain, which is caused by ongoing inflammation and damage of non-neuronal tissues (Haleem, 2019; Fitzcharles et al., 2021). However, unlike acute pain which carries a protective value, chronic pain serves no obvious beneficial function, and often causes serious mental disorders, like persistent anxiety and depression (Bushnell et al., 2013; Cohen et al., 2021).

Usual treatments for chronic pain conditions are divided into classical pharmacological treatments and non-pharmacological therapies. Pharmacological treatments include non-steroidal anti-inflammatory drugs (NSAIDs), topical agents like capsaicin, antidepressants [selective serotonin reuptake inhibitors (SSRIs), serotonin-norepinephrine reuptake inhibitors (SNRIs) and tricyclics], anticonvulsants and opioids (Fitzcharles et al., 2021). NSAIDs and SNRIs (e.g., duloxetine) are often used to treat chronic low back pain of unknown causes. Low-dose tricyclics, SNRIs and anticonvulsants are always applied in fibromyalgia treatment (Fitzcharles et al., 2021). Topical patches and injectable formulations containing capsaicin have shown success in clinical applications to treat neuropathic pain and osteoarthritis through persistent desensitization of peripheral nociceptive receptors (Arora et al., 2021). Whereas weak opioid analgesics (e.g., tramadol) and strong opioids (e.g., morphine) are only recommended in some refractory central and peripheral neuropathic pain conditions (Szok et al., 2019). Although some drugs, like SSRIs and opioids, are prescribed strictly in doses known to relieve pain, their long-term application in chronic pain is still largely unknown as a result of drug resistance, undesirable side effects, physiologic dependence, and risk of addiction (Ambrose and Golightly, 2015). Despite the lack of a clear mechanism, antidepressants, like fluoxetine, have a considerable effect on the treatment of chronic pain and anxiety (Bandelow et al., 2017; Haleem, 2019). However, not all antidepressant drugs produce the desired analgesic effect under different chronic pain conditions (Haleem, 2019). Further studies are needed to improve the treatment efficacy of these antidepressant drugs. Non-pharmacological therapies are also becoming important components for the clinical management of chronic pain, such as cognitive behavioral therapy, physical exercise, and electrical/magnetic stimulation (Ambrose and Golightly, 2015; Ji et al., 2018; Fitzcharles et al., 2021), however, the application and the efficacy of these therapies are dependent on the specific diseases restrictively.

Preclinical animal studies have reported depression- and anxiety-like behaviors in different chronic pain models (Yamauchi et al., 2022). These findings suggest that chronic pain causes plastic changes in neural circuits and leads to the experience of negative emotions. Meanwhile, it is also widely accepted that one of the main causes of chronic pain results from central nervous sensitization. According to current literature, the local synaptic plasticity in the central nervous system (CNS), such as the anterior cingulate cortex (ACC), plays a critical role in chronic pain and injury-related anxiety (Apkarian et al., 2011; Bliss et al., 2016; Zhuo, 2016; Kuner and Flor, 2017). It was reported that the enhancement of pre-/postsynaptic plasticity in the ACC promoted chronic pain and its related anxiety, respectively, in neuropathic and inflammatory pain models (Koga et al., 2015). Considering the importance of synaptic plasticity, a better understanding of the mechanisms underlying the interaction between neuronal plasticity and chronic pain/anxiety is fundamental to our understanding of chronic pain.

Serotonin (5-HT), as a key neurotransmitter, contributes to multiple physiological functions such as pain sensation, inflammation, cognition, and emotions–especially anxiety and depression. Although the percentage of 5-HT neurons in the mammalian brain is small (less than 0.1%), they collectively innervate most of the brain regions and play various critical roles by the broadly expressing receptors (Okaty et al., 2019). In the human brain, like in other species, the spinal cord and many pain/anxiety-related brain regions, such as the thalamus, frontal cortex, amygdala, and brainstem, are innervated by 5-HT neurons and express one or multiple types of 5-HT receptors (Charnay and Leger, 2010). Given the critical roles of 5-HT in the CNS, it is not surprising that 5-HT and its receptors have long been recognized as key modulators in pain processing and potential targets for pain treatment (Liu et al., 2020). The 5-HT system exerts different regulations of pain perception and anxiety in different ways (Charnay and Leger, 2010; Ji et al., 2018; Liu et al., 2020). On the one hand, 5-HT can promote or inhibit pain perception through different neural circuits, such as the spinal descending facilitatory/inhibitory pathways. On the other hand, in the same CNS regions, 5-HT can also exert excitatory/inhibitory functions depending on the diversity of its receptors and respective downstream signaling pathways. Moreover, the 5-HT system has been shown to change synaptic transmission and plasticity diversely in the central nervous areas, such as the ACC and the spinal cord, both of which are closely associated with chronic pain and injury-related anxiety (Li and Zhuo, 1998; Tian et al., 2017). Clinically, many agonists/antagonists of 5-HT receptors (e.g., the 5-HT_1*A*_ receptor agonist buspirone and the selective 5-HT_3_ receptor antagonist alosetron), SSRIs (e.g., fluoxetine and paroxetine) and SNRIs (e.g., duloxetine and venlafaxine) have been used to treat different pain- and anxiety-related conditions as the first-line drugs (Jann and Slade, 2007; Haleem, 2019; Liu et al., 2020; Cohen et al., 2021). For example, alosetron is the only FDA (US Food and Drug Administration)-approved drug for irritable bowel syndrome (Paredes et al., 2019). Fluoxetine is reported to be effective in the treatment of tension headaches (Walker et al., 1998). However, the therapeutic mechanisms of these drugs are still unclear.

The main purpose of this review is to summarize the neural pathways and receptor distribution of the 5-HT system associated with chronic pain and injury-related anxiety, and describe its pathophysiologic roles and synaptic mechanisms in the CNS, including synaptic transmission and plasticity. This review will guide future research efforts in identifying new therapeutic methods for the treatment of chronic pain and injury-related anxiety.

## Overview of serotonergic projections throughout pain- and anxiety-related pathways

Serotonin neurons are distributed in very few brain regions. Previous studies have indicated that 5-HT neurons in the rodent brain were intensively distributed along the midline of the raphe nuclei and its surrounding nuclei of the brainstem. These serotonergic nuclei can be divided into two groups generally, including the rostral and caudal nuclei. The rostral nucleus in the midbrain and rostral pons sends ascending projections to the forebrain and the brainstem regions, and descending projections to the spinal cord (Hornung, 2010). Their ascending projections cover the majority of the brain, including the cingulate cortex, midbrain, thalamus, hippocampus, cerebellum, insular cortex (IC), prefrontal cortex (PFC), parietal, and occipital cortical regions (Charnay and Leger, 2010; Hornung, 2010; Gogolla, 2017; Cortes-Altamirano et al., 2018). Whereas, the caudal nucleus in the caudal pons and medulla oblongata mostly forms descending projections to the spinal cord gray matter [including the dorsal horn (DH), ventral horn, and intermediate area], and innervates distinct regions in the brainstem and forebrain upwardly (Hodges and Richerson, 2008, 2010; Hornung, 2010; Ghosh and Pearse, 2015).

**FIGURE 1:**
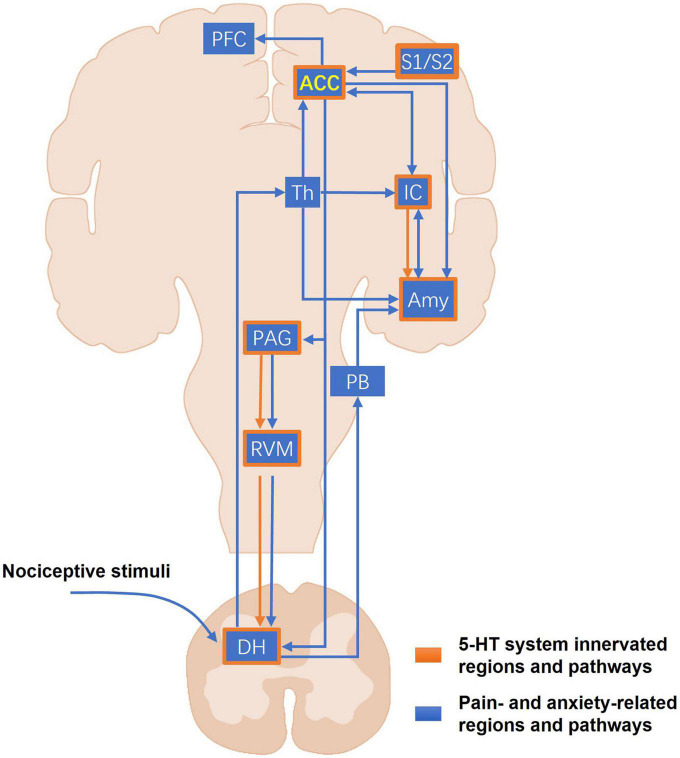
The major pain- and anxiety-related pathways of the 5-HT system. The blue blocks and lines represent the major pain- and anxiety-related regions and projections in the CNS. The peripheral nociceptive stimuli first arrive at the DH. Then the ascending nociceptive inputs can be sent into the ACC through the thalamus and other brain regions involved in the emotional process, such as the amygdala and the IC. Another part of nociceptive inputs can be conveyed to the amygdala through the PB. As a higher center of pain perception and anxiety, the ACC marked in yellow forms the unilateral or reciprocal projections with many other important cortical regions, including the amygdala, the IC, the PFC, and the somatosensory cortices that mediate sensory modulation, anxiety, fear, and memory. In the descending pathways, the ACC sends their projections directly or indirectly to the DH, allowing cortical neurons to directly modulate the sensory input into the CNS. The orange frames and lines mark the known CNS regions and projections where the 5-HT system participates in the modulation of pain and pain-related anxiety. The 5-HT system distributes widely and almost innervates most of the core regions for pain and anxiety, such as the ACC, IC, amygdala, and the PAG-RVM-DH descending pathway. ACC, anterior cingulate cortex; Amy, amygdala; DH, dorsal horn; IC, insular cortex; PAG, periaqueductal gray; PB, parabrachial area; PFC, prefrontal cortex; RVM, rostroventral medulla; S1, primary somatosensory cortex; S2, secondary somatosensory cortex; Th, thalamus.

Brain-imaging studies in humans have provided crucial information about the brain regions involved in different types of pain (Bushnell et al., 2013). The ACC and other brain regions, including the IC, primary somatosensory cortex (S1), secondary somatosensory cortex (S2), PFC, thalamus, and cerebellum, are activated by various noxious stimuli (Apkarian et al., 2005). In addition, accumulated studies in humans have shown that certain brain regions, such as the ACC, IC, and amygdala, associated with negative emotions in chronic pain patients can be activated during noxious stimulation and be altered in structure, activity, or connectivity, indicating the comorbidity of chronic pain and mental disorders (Bushnell et al., 2013; Denk et al., 2014; Bliss et al., 2016). Coincidentally, many 5-HT innervated brain and spinal regions are also involved in the regulation of pain perception and anxiety (Figure 1). Under physiological conditions, the noxious stimulation first activates peripheral nociceptive afferent fibers. Incoming action potentials trigger a release of excitatory neurotransmitters and activate ascending neurons in the DH. In the brain, the peripheral noxious inputs are conveyed from the DH to ACC through the thalamic relays. Additionally, nociceptive information can be relayed to the ACC through the amygdala which receives spinal sensory inputs through the parabrachial area (PB) in rodents. The ACC, IC, and amygdala innervated by 5-HT neurons are well-known to participate in different pain processes and the generation of emotional disorders, such as anxiety and fear, both in humans and laboratory animals (Bliss et al., 2016; Gogolla, 2017). The ACC can also directly modulate the sensory input via ACC-IC and ACC-PFC projections, and facilitate pain sensitization via ACC-DH and ACC-PAG (Periaqueductal gray)-RVM (Rostral ventromedial medulla)-DH descending circuits directly or indirectly (Bliss et al., 2016). In a human brain imaging study, acute tryptophan depletion (ATD) via the reduction of 5-HT in the human brain was shown to alter the activated volumes of bilateral S1 and S2 and decreased volunteers’ pain threshold in response to electrical stimuli (Wang et al., 2010). Another recent study demonstrated that the activation of S1-ACC projections increased the response to noxious stimuli in rat ACC neurons (Singh et al., 2020). As a key part of nociceptive descending modulation, the 5-HT system exerts both descending facilitation and inhibition on pain perception in the spinal cord depending on acute or chronic pain states and its receptor types (Zhuo, 2008; Cortes-Altamirano et al., 2018). The 5-HT neurons in the RVM are also recognized to provide the major descending serotonergic projections to the spinal cord as an important contributor to chronic pain facilitation (Wei et al., 2010; Cai et al., 2014). In mice, the ACC, IC, and the amygdala have all been shown to be closely associated with anxiety states (Barthas et al., 2015; Koga et al., 2015; Gehrlach et al., 2019). Barthas et al. (2015) found that selective optogenetic activation of pyramidal neurons of the ACC in mice induced anxiety-like behaviors–such as burying more marbles. Another recent study showed that the projections from IC to central amygdala (CeA) mediated anxiety-related behaviors in mice. Silencing the IC-CeA pathway by optogenetics could increase the time in the open arm and induce an anxiolytic response in the elevated plus maze (EPM) test (Gehrlach et al., 2019). Therefore, it is clear that the 5-HT system is anatomically and functionally integrated with the pain/anxiety-related pathways, implicating the potential roles of the 5-HT system in the regulation of pain and injury-related anxiety.

## 5-HT receptor distribution and function across pain/anxiety-related brain regions

Currently, 7 families of 5-HT receptors (5-HT_1_ to 5-HT_7_) have been identified, with at least 14 subtypes in mammals (Barnes and Sharp, 1999). Among these receptors, only 5-HT_3_ receptors belong to the ionotropic receptors which are permeable to cations in the activated state, and all the others belong to G protein-coupled receptors (GPCRs). As the most injury-related receptors, 5-HT_1/2/3/7_ receptors intensively express in the ACC, the PFC, the amygdala, and the DH (Charnay and Leger, 2010; Cortes-Altamirano et al., 2018). For example, different 5-HT_1_ receptor agonists inhibit the nociceptive tail-flick reflex when administered into the spine (Eide et al., 1990). 5-HT_4/5/6_ receptors are also widely distributed in the pain-/anxiety-related cortical and subcortical regions, such as the PFC and thalamus (Charnay and Leger, 2010; Liu et al., 2020). Godínez-Chaparro et al. (2012) described the pronociceptive role of spinal 5-HT and 5-HT_4/6_ receptors in the long-term secondary mechanical allodynia and hyperalgesia induced by formalin in rats. Additionally, 5-HT_4/5/6_ receptors are found to participate in the modulation of chronic pain in the ventrolateral orbital cortex (Liu et al., 2020). Although 5-HT receptors show much overlapped expression in the central and peripheral nervous system, their intracellular localization is not completely consistent at the subcellular level (Borroto-Escuela et al., 2021). Thus, it is important to elucidate the subcellular distribution of 5-HT receptor expression for a better understanding of their functions in pain and anxiety.

**FIGURE 2:**
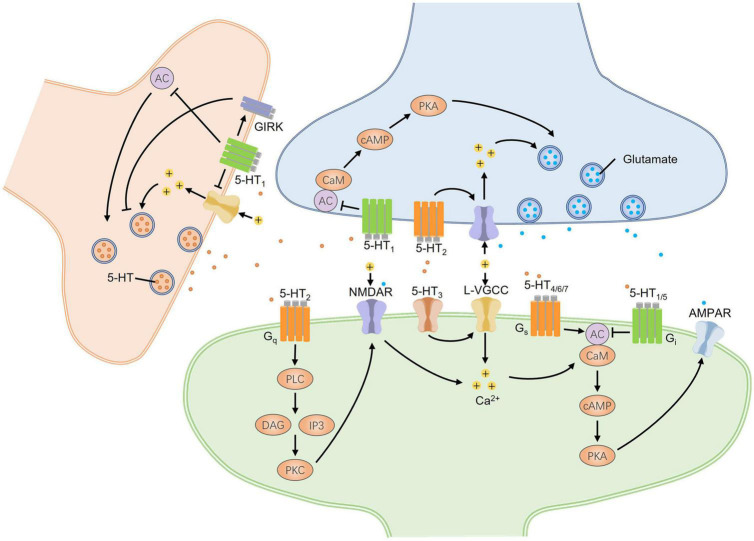
The bidirectional regulation of synaptic transmission mediated by signaling pathways of 5-HT receptors in the CNS. Different presynaptic and postsynaptic 5-HT receptors undergo excitatory and inhibitory synaptic changes. Three groups of G-protein coupled 5-HT receptors, including G_s_/G_q_/G_*i*_-protein coupled groups, mainly mediate intracellular canonical cascades. 5-HT_1/5_ receptors coupled with G_*i*_-protein downregulate synaptic excitability, while 5-HT_4/6/7_ receptors coupled with G_*s*_-protein upregulate that by affecting the activity of AC and the downstream PKA signaling pathway. Both AC and PKA are critical for the presynaptic enhancement of glutamate release and the postsynaptic potentiation of AMPA receptors. 5-HT_2_ receptors coupled with G_*q*_-protein can trigger the PKC signaling pathway that promotes the phosphorylation of NMDA receptors and neural hyperexcitation by producing Ca^2+^ inflows. At postsynaptic sites, 5-HT_3_ receptors, as the cation channels, can induce neural depolarization and open L-VGCC to increase intracellular Ca^2+^ concentrations which can also activate AC by binding CaM. The presynaptic expression of 5-HT_1_ autoreceptors can decrease presynaptic 5-HT release by directly inhibiting the activity of AC, closing Ca^2+^ channels, or opening GIRKs to produce presynaptic depolarization. Taken together, 5-HT receptors have bidirectional effects on the regulation of synaptic transmission. AC, adenylyl cyclase; CaM, calmodulin; cAMP, cyclic adenosine monophosphate; DAG, diacylglycerol; GIRK, G-protein-gated inwardly rectifying potassium channels; IP3, inositol 1,4,5 triphosphate; L-VGCC, L-type voltage-gated calcium channel; PKA, protein kinase A; PKC, protein kinase C; PLC, phospholipase C.

According to the difference of coupled G-proteins, 5-HT GPCRs can be classified into 3 groups, including G_*s*_/G_*q*_/G_*i*_-protein coupled groups (Figure 2). Both 5-HT_1_ and 5-HT_5_ receptor families coupled with G_*i*_ proteins inhibit the activity of adenylyl cyclase (AC), decreasing the intracellular cyclic adenosine monophosphate (cAMP) levels and causing canonical second messenger cascades. On the contrary, 5-HT_4_, 5-HT_6_, and 5-HT_7_ receptor families coupling with G_*s*_ subunit can activate AC and increase cAMP production. The increased cAMP next facilitates L-type calcium channels and induces a slow depolarization of membrane potential, exerting an excitatory effect on the neurons (Sharp and Barnes, 2020). The 5-HT_2_ receptor family belongs to the G_*q*_-protein coupled group, which activates phospholipase C (PLC) and then degrades phosphatidylinositol 4,5 bisphosphate (PIP2) into the production of inositol 1,4,5 triphosphate (IP3) and diacylglycerol (DAG) (Figure 2). In addition, the metabotropic 5-HT receptors can also recruit many other non-canonical signaling pathways, such as extracellular signal-regulated kinase (ERK) signaling pathway, phospholipase A2, and β-arrestin coupled Src/Akt cascades (Sharp and Barnes, 2020). Furthermore, adjacent membrane proteins and allosteric modulators of metabotropic 5-HT receptors have been reported to regulate the function of 5-HT receptors via altering cellular localization or molecular structure to trigger a specific downstream reaction (Sharp and Barnes, 2020; Popova et al., 2022). Another mechanism regulating the function of the 5-HT receptor is RNA editing. The adenosine to-inosine RNA editing mechanism can generate multiple isoforms of 5-HT receptor that differ in G-protein coupling efficacy and constitutive activity. For example, five adenosine sites of the 5-HT_2C_ receptor can be flexibly converted to inosine by RNA editing. Fully edited transcripts and partially edited transcripts differ from non-edited receptors in their reduced G-protein coupling and decreased serotonergic potency (O’Neil and Emeson, 2012). The 5-HT_3_ receptor family belongs to a ligand-gated ion channel permeable to Ca^2+^, Na^+^, and K^+^, mediating an inward current and depolarizing the neurons. This depolarization could trigger various second messenger signaling pathways, regulating the surrounding synaptic plasticity and fast synaptic transmission (Cortes-Altamirano et al., 2018). The complexity of 5-HT receptor signaling pathways and some special regulatory manners remind us that 5-HT might exert diverse synergistic and antagonistic effects on pain perception and anxiety.

## Contributions of the 5-HT system to chronic pain

In both the peripheral and central nervous systems, 5-HT displays different roles in chronic pain regulation (Liu et al., 2020). 5-HT acts with both nociceptive and antinociceptive or even biphasic effects depending on the type of receptor, amount of substance, and anatomical region (Paredes et al., 2019). In humans, an acute tryptophan depletion (ATD) in the brain can alter pain perception (Martin et al., 2017; Trotter et al., 2022). A tryptophan-free amino acid drink was administered to induce acute central depletion of 5-HT due to the competitive uptake of large neutral amino acids across the blood-brain barrier, without any impact on peripheral 5-HT function (Trotter et al., 2022). Martin et al. (2017) found that ATD significantly reduced the pain threshold and tolerance in response to a heat thermode. Another recent study demonstrated that acute depletion of central 5-HT levels enhanced hedonic ratings of affective touch, and reduced the tolerance to cold pain in humans (Trotter et al., 2022). In laboratory animals, the 5-HT system also plays a critical role in chronic pain regulation. For example, acute and chronic pain is associated with traumatic brain injury (TBI). Selective spinal 5-HT fiber depletion with 5,7-dihydroxytryptamine (5,7-DHT) reduced hypersensitivity in the mice with mild TBI (mTBI). Normally, 5-HT promoted nociceptive sensitization directly through 5-HT_3_ receptors and indirectly through the upregulation of chemokine signaling after mTBI (Sahbaie et al., 2019). A similar behavioral result was reported in the rat spinal nerve ligation (SNL) model (Rahman et al., 2006). However, in the rat paw pressure test, the intrathecal injection of 5-HT produced significant antinociceptive effects, while peripheral intraplantar 5-HT could be nociceptive and induce mechanical allodynia because of the increase in inflammatory response (Sufka et al., 1992; Bardin et al., 2000). In another formalin rats test, the intrathecal administration of 5-HT showed a biphasic effect on pain modulation. 5-HT inhibited the aversive responses when administered at a low dose (0.1 nmol/rat), but facilitated them at a high dose (1 nmol/rat). Further results suggest that 5-HT suppressed formalin-induced nociception in the spinal cord via the 5-HT_1A_ receptor and facilitated it via the 5-HT_3_ receptor (Oyama et al., 1996). These actions of 5-HT system reveal the complexity of its mechanisms in the modulation of nociception, which relies on the diversity of 5-HT receptors both at the periphery, and within the CNS (Viguier et al., 2013).

In the spinal cord, 5-HT can be pronociceptive or antinociceptive depending on its receptor subtypes activated. Generally, activation of the 5-HT_1A_, 5-HT_1B_, 5-HT_1D_, and 5-HT_7_ receptors tends to be antinociceptive, whereas the 5-HT_2A_ and 5-HT_3_ receptors tend to promote nociception (Ossipov et al., 2014). Both selective 5-HT_1A_ receptor antagonists WAY-100635 and selective 5-HT_1B/1D_ receptor antagonists GR-127935 can counteract the antiallodynic effect induced by spinal 5-HT in neuropathic pain models (Avila-Rojas et al., 2015). Otherwise, 5-HT_2A_ receptor is considered to be pronociceptive in the spinal cord. The 5-HT_2A_ receptor could promote spinal hyperexcitability and neuropathic pain by affecting pain-related protein expression (Liu et al., 2020). One previous study showed that infraorbital nerve injury affected RNA editing efficiency, changing the proportional presence of the 5-HT_2C_ receptor isoforms in the rat cervical spinal cord. The post-injury change increased the expression of less edited receptor isoforms and reduced the expression of more edited receptor isoforms, making spinal 5-HT receptors more sensitive to 5-HT. 5-HT would then activate the brainstem-spinal descending inhibitory pathway to suppress nociceptive signals from primary afferent neurons to the spinal trigeminal nucleus caudalis. This modulation of mRNA editing could be an adaptive mechanism that maintains the input-output balance of 5-HT system and functions in response to serotonergic dysfunction under some pathological conditions, such as 5-HT depletion or surgical stress (Nakae et al., 2008). For 5-HT_3_ receptors, a study showed that the spinal application of 5-HT_3_ receptor antagonist ondansetron could block the descending facilitation and completely inhibit mechanical hyperalgesia and thermal allodynia in the rat SNL model (Dogrul et al., 2009). Intrathecal administration of selective 5-HT_4_ (GR-113808) and 5-HT_6_ (SB-258585) receptor antagonists decreased tactile allodynia in L5/L6 spinal nerve ligation rats. Selective 5-HT_4_ (ML-10302) and 5-HT_6_ (WAY-208466) receptor agonists prevented the antiallodynic effect of the antagonists. These results suggest that spinal 5-HT_4_ and 5-HT_6_ receptors also participated in the maintenance of neuropathic pain (Pineda-Farias et al., 2017). 5-HT_7_ receptors in the spinal cord also participate in the processing of antinociception. Brenchat et al. (2010) found a significant increase of 5-HT_7_ immunoreactivity on the ipsilateral side of the spinal cord after nerve injury. Gautier et al. (2017) accomplished lentiviral vector-driven inhibition of 5-HT synthesis specifically within bulbar 5-HT neurons projecting into the DH. Their study demonstrated that these descending serotonergic projections exerted an antinociceptive action in the neuropathic pain models. Additionally, intraplantar administration of a selective 5-HT_7_ receptor antagonist (SB-269970) completely blocked the morphine-induced antinociception in a time-dependent manner, indicating the analgesic effect of the spinal 5-HT_7_ receptor (Dogrul and Seyrek, 2006). Meanwhile, Yang et al. (2014) found that the spinal 5-HT_7_ receptor played a significant inhibitory role in descending serotonergic modulation in inflammatory pain induced by formalin but not carrageenan. However, in the carrageenan model, the spinal 5-HT_3_ receptor predominantly mediated pronociceptive effects (Yang et al., 2014). Besides, the contrary results of these receptors mentioned above ought to get more attention. More studies are required to precisely clarify the roles of spinal 5-HT receptors in chronic pain.

At the supraspinal level, the 5-HT system also contributes to the regulation of chronic pain. The PAG-RVM-DH circuit underlies the pain descending regulation (Zhuo, 2008). As reported, the 5-HT_7_ receptor agonist AS-19 exerted a dose-dependent antinociceptive effect when administered into the PAG in the rat chronic constrict injury (CCI) model. AS-19 microinjection significantly elevated the mechanical withdrawal threshold values, but SB-269970 pretreatment attenuated the antihyperalgesic effect of AS-19 (Li et al., 2014). The Wei et al. (2010) study revealed that selective ablation of descending 5-HT neurons in the RVM with regional shRNA interference (RNAi) could reduce formalin-induced persistent pain and injury-induced allodynia and hyperalgesia in rats. Repeated activation of RVM 5-HT neurons by optogenetics also decreased both mechanical and thermal pain thresholds and produced sensitized pain behaviors for up to 2 weeks in tryptophan hydroxylase 2 (TPH2)-Channelrhodopsin 2 (ChR2) transgenic mice, but not in wild type mice (Cai et al., 2014). Therefore, the 5-HT in this descending pathway is indeed critical for persistent pain facilitation. As a higher brain region, the ACC is often recognized as a cortical regulatory center of pain perception (Chen et al., 2021). The neuroplasticity, particularly the postsynaptic long-term potentiation (LTP), in ACC excitatory synapses is highly related to chronic pain. The postsynaptic LTP in the ACC is triggered by the activation of phosphorylated N-methyl-D-aspartate (NMDA) and the increase of α-amino-3-hydroxy-5-methyl-4-isoxazolepropionic acid (AMPA) receptor function, which underlies the unpleasant experience of chronic pain (Bliss et al., 2016). The Tian et al. (2017) study showed that 5-HT could inhibit the glutamatergic synaptic transmission in the ACC through the 5-HT_1A_ receptor. Specific activation of the 5-HT_7_ receptors in the ACC by bath application of a 5-HT_7_ receptor agonist 5-carboxamidotryptamine (5-CT) could restore normal dendritic integration and produce analgesic effects in the CCI mice. This effect was completely blocked by SB-269970 (Santello and Nevian, 2015). Thus, 5-HT receptors may regulate chronic pain by affecting adjacent glutamate receptors and changing excitatory synaptic transmission in the ACC. The ACC can also form reciprocal connections with other cortical regions, such as IC and amygdala, which are also critical for pain perception (Bliss et al., 2016). Human brain imaging studies supported the important roles of IC in pain perception, especially pain-related emotions (Bushnell et al., 2013). It has been reported that both NMDA and AMPA receptors were upregulated in the mouse IC after peripheral nerve injury (Qiu et al., 2013, 2014). This long-term alteration can enhance presynaptic neurotransmitter release and postsynaptic responsiveness in the IC, which probably contributes to the formation of chronic pain and injury-related anxiety (Zhuo, 2016). Moreover, there is some evidence to indicate that the activation of 5-HT_1A_ receptor in the IC mediates chronic stress-induced visceral hypersensitivity (Sun et al., 2016). Similarly, the effects of the 5-HT system on chronic pain modulation also exist in the amygdala. 5-HT and 5-HT_2C_ receptors in the amygdala could significantly affect neuropathic and inflammatory pain-related responses and behaviors (Ji et al., 2017; Zhang et al., 2018). For example, direct injecting 5-HT into the mice CeA suppressed both the spontaneous pain behaviors and hyperalgesia induced by formalin injection (Zhang et al., 2018). Whereas, Ji et al. (2017) found that 5-HT_2C_ receptor knockdown in the basolateral amygdala (BLA) blocked the increase in excitatory transmission from BLA to CeA and increased sensory thresholds in SNL rats, implicating that 5-HT_2C_ receptor in the BLA mediated neuropathic nociception. It was also reported that the microinjection of SSRI paroxetine into the S1, a key cortical region of pain sensation, significantly attenuated thermal hyperalgesia in mice, suggesting that 5-HT could enhance chronic pain sensation in the S1 (Matsuzawa-Yanagida et al., 2008).

In summary, it is clear that the 5-HT system in the spinal cord and many brain regions plays an important role in chronic pain regulation. To date, a considerable number of studies have focused primarily on the nociceptive or antinociceptive actions of 5-HT_1/2/3/7_ receptors in the spinal cord and brain, which provides us with promising pain-relieving targets and novel treatment strategies for chronic pain. However, more recent efforts have been focused on the actions of other receptor subtypes, like 5-HT_4/5/6_ receptors, and the roles of 5-HT receptors in the higher brain regions. Further studies, especially those at synaptic levels, may generate new ideas in the treatment of chronic pain.

## Contributions of the 5-HT system to injury-related anxiety

In humans, it is well-known that anxiety can enhance pain perception, and the experience of chronic pain can lead to persistent anxiety (Bliss et al., 2016). The behavioral analysis in a recent study demonstrated that 5-HT depletion–whether it was at the periphery or in the brain–presented reduced anxiety-like behaviors in rodents (Mosienko et al., 2012; Sbrini et al., 2022). They used *Tph1/Tph2*-deficient mice to evaluate the impact of 5-HT depletion at the peripheral or in the brain, respectively, on mouse behavior. Both *Tph1^–/–^* and *Tph2^–/–^* mice exhibited decreased anxiety-like behaviors in the EPM. These results indicate the key role of peripheral or central 5-HT in anxiety. Although the exact mechanism of SSRIs is still unclear, they are now widely accepted as the first-line agents for the treatment of anxiety disorders (Calhoon and Tye, 2015). Matsuzawa-Yanagida et al. (2008) found that local injection of SSRI paroxetine into different cortical regions, such as the cingulate cortex or BLA, reduced anxiety-like behaviors in the EPM and the light-dark test in the mice neuropathic pain model. Their results proved that SSRIs were effective for treating anxiety associated with neuropathic pain, and had anxiolytic effects by acting on different brain regions. In another study, anxiety-like behaviors were also altered in 5-HT_1A_ or 5-HT_2A_ receptor knockout mouse lines (Ramboz et al., 1998; Weisstaub et al., 2006). 5-HT_1A_ receptor global knockout mice displayed less exploratory activity and more anxiety-like behaviors in the open field and EPM tests, while the global disruption of 5-HT_2A_ receptor transcription in genetically modified mice induced anxiolytic behaviors, including more exploration in the center or open arm areas. In consideration of all of these findings, it is clear that 5-HT functions differently in the regulation of different anxiety states. How it functions is dependent on many factors, such as the local levels of 5-HT, the receptor subtypes and their sensitivity, the action regions, and the specific physiological/pathological conditions.

According to our findings, the ACC presynaptic LTP (pre-LTP) in excitatory transmission shows a close relationship with injury-induced anxiety (Koga et al., 2015). The kainate receptor-mediated presynaptic LTP in the ACC contributed to long-lasting anxiety induced by chronic pain, which also required the involvement of AC and a protein kinase A (PKA). The application of 5-HT could significantly inhibit excitatory synaptic transmission, and decrease presynaptic glutamate release of the ACC via the 5-HT_1A_ receptor in brain slices of mice (Tian et al., 2017). Barthas et al.’s (2015) results also confirmed the key role of the excitatory neurons of the ACC in anxiety. Recently, a new study described that regular aerobic exercise had significant effects in relieving pain and concomitant anxiety in the chronic inflammatory pain model. Regular aerobic exercise could increase 5-HT release and attenuate pain-induced LTP occlusion in the ACC through the 5-HT_1A_ and 5-HT_7_ receptors (Zhou et al., 2022). Therefore, 5-HT may be modulating injury-related anxiety by directly or indirectly changing the pre-LTP of excitatory transmission in the ACC.

In addition, the IC is also identified as an important cortical region involved in anxiety (Zhuo, 2016; Gogolla, 2017). Some clinical results confirmed the correlation between IC activity and anxiety (Etkin and Wager, 2007). Hyperactivation in the IC and amygdala of patients was frequently found in subjects with a social anxiety disorder by functional magnetic resonance imaging and positron emission tomography (PET). Some other PET studies showed the decreased binding potential of 5-HT_1A_ and 5-HT_2A_ receptors in the IC of patients and marmosets diagnosed with social anxiety disorder and trait anxiety, respectively (Santangelo et al., 2019; Ju et al., 2020). The synaptic plasticity in the IC mainly depends on the mediation of glutamate receptors (Zhuo, 2016). Specifically, the electrophysiological results showed that the kainate receptors contributed to fast synaptic transmission in the IC. After the blockade of AMPA and NMDA receptors, kainate receptor-mediated excitatory postsynaptic currents (EPSCs) were observed (Koga et al., 2012). These findings suggest it is possible that presynaptic kainate receptors in the IC participate in the pre-LTP induction and promote persistent anxiety by a common mechanism as we have seen in the ACC.

The amygdala is another important brain region for emotional anxiety, as it can receive the serotonergic projections from the raphe nucleus and connect with the ACC and IC simultaneously (Gross and Hen, 2004; Hornung, 2010; Bliss et al., 2016). This anatomical connection implies the involvement of the amygdala 5-HT system in anxiety. A recent study showed that the serotonergic projections from dorsal raphe nuclei to the amygdala promoted anxiety-like behavior, causing a significant decrease of center time in the open field test (Ren et al., 2018). It has been also reported that 5-HT affects synaptic transmission in the amygdala. Local 5-HT_2C_ receptor knockdown with the stereotaxic injection of 5-HT_2C_ receptor shRNA AAV vector in the BLA could significantly decrease anxiety-like behaviors in the EPM and increase the mechanical withdrawal thresholds in the SNL rats (Ji et al., 2017).

Taken together, it is clear that the 5-HT system promotes or inhibits injury-related anxiety according to its receptor subtypes in different brain regions–as seen with an anxiolytic effect in the ACC, but an anxiogenic effect in the amygdala. 5-HT_1/2/7_ receptors within the ACC, IC, and amygdala seem to be promising targets for anxiety treatment. Further research into the mechanisms of the 5-HT system in the brain will help us explore new methods for treating anxiety.

## Synaptic mechanisms of the 5-HT system in chronic pain and injury-related anxiety

To date, there are several prevalent mechanisms to explain the pathogeny of chronic pain and its related emotional disorders. One prevalent mechanism is that neuroinflammation drives central sensitization in the peripheral and central nervous systems. A characteristic feature of neuroinflammation is the activation of glial cells, such as microglia and astrocytes in the CNS. This activation could promote the local release of proinflammatory cytokines and chemokines, such as transforming growth factor (TGF), brain-derived neurotrophic factor (BDNF), and interleukin (Ji et al., 2018). Meanwhile, central cytokines and chemokines are recognized as powerful neuromodulators and play a sufficient role in neuropathic pain (Ding et al., 2020). Another mechanism is the interaction between 5-HT and other neurotransmitters. 5-HT is known to regulate the release of many neurotransmitters, such as GABA, glutamate, dopamine, and noradrenaline. The 5-HT system also interacts with peptidergic transmissions, endocannabinoid systems, and glial factors (De Deurwaerdere and Di Giovanni, 2021). For example, in the spine of neuropathic pain models, both the activation of 5-HT_3_ receptors and 5-HT_7_ receptors can recruit GABA into the analgesic effects (Liu et al., 2020). This complexity of the interaction confers 5-HT multiple potential roles in both chronic pain and anxiety. Although there are many causes leading to chronic pain and anxiety, the primary and direct cause is still the long-term sensitization of the peripheral or central nervous system. This long-term nervous system sensitization mainly results from the change in synaptic transmission and plasticity. Here, we will focus on the roles of the 5-HT system in synaptic transmission and plasticity associated with chronic pain and anxiety.

Synaptic modulation mediated by 5-HT is an important mechanism underlying its physiological function. Previous studies have shown that 5-HT can produce both excitatory and inhibitory modulation at spinal glutamatergic synapses, consistent with the biphasic modulatory effects of 5-HT on spinal nociceptive transmission and behavioral reflexes (Li and Zhuo, 1998). To date, there is much more evidence supporting the diverse synaptic modulation of the 5-HT system.

### Effects of the 5-HT system on excitatory and inhibitory synaptic transmission

As we know, the DH is the first-order center for pain transmission. Hori et al. (1996) and Lopez-Garcia and King (1996) studies showed that 5-HT could bidirectionally affect spinal synaptic transmission by acting on presynaptic or postsynaptic receptors. Li and Zhuo (1998) found that 5-HT could transform silent glutamatergic synapses into functional ones in the rat spinal cord. In this study, they demonstrated a dose-dependent and biphasic modulation of 5-HT at spinal excitatory synapses. For example, 5-HT at a high dose (50 μM) produced inhibition of EPSCs, while a low dose (5 μM) of 5-HT induced facilitation of fast EPSCs in rat spinal cord slices (Li and Zhuo, 1998). This synaptic mechanism may contribute to plastic changes in nociception after tissue or nerve injury. Recently, Tian et al. (2017) investigated the role of 5-HT on glutamatergic neurotransmission in the ACC. Bath application of 5-HT produced dose-dependent inhibition of different EPSCs significantly. Meanwhile, the increased paired-pulse ratio (PPR) indicated the serotonergic presynaptic inhibitory effect. Finally, the application of the 5-HT_1A_ receptor antagonist NAN-190 significantly reduced serotonergic postsynaptic inhibition and abolished presynaptic inhibition, indicating that both presynaptic and postsynaptic 5-HT receptors contributed to this inhibitory synaptic modulation (Tian et al., 2017). This inhibition of the ACC potentiation should be analgesic and anxiolytic in animal models of chronic pain (Zhuo, 2014; Koga et al., 2015). The collective results indicate that the 5-HT system has a significant effect on synaptic transmission through both presynaptic and postsynaptic ways.

Serotonin regulates synaptic transmission by acting on its presynaptic and postsynaptic receptors (Figure 2). Postsynaptic application of G-protein inhibitor GDP-β-S abolished the facilitatory effect induced by 5-HT in the spine, implying that postsynaptic 5-HT GPCRs were critical for the enhancement of the response to noxious stimuli (Li and Zhuo, 1998). In addition, postsynaptic Ca^2+^-dependent processes were required for 5-HT-induced facilitation. 5-HT-induced facilitation disappeared by postsynaptic application of the Ca^2+^ chelator BAPTA, indicating the essential role of postsynaptic increased Ca^2+^ (Zhuo, 2000). Another study showed that postsynaptic activation of protein kinase C (PKC) was required for synaptic potentiation induced by 5-HT in the spinal cord. Moreover, the PKC-mediated activation of silent synapses was also dependent on the interactions between AMPA receptor subunits GluR2/3 and PDZ-domain-containing proteins (Li et al., 1999). The further study also found that in the adult DH, co-application of AC activator forskolin and 5-HT could induce long-lasting enhancement, including the recruitment of functional AMPA receptor-mediated responses. Ca^2+^-sensitive, calmodulin-regulated adenylyl cyclases (AC1 and AC8) were necessary for this enhancement (Wang and Zhuo, 2002). Besides, Aira et al. (2013) found that activation of 5-HT_2B_ receptors led to the enrichment of postsynaptic PKC_γ_, phosphorylated NMDA receptor subunit, and neural hyperexcitation in the DH. 5-HT could modulate the postsynaptic transmission to affect pain perception by changing the distribution and activity of associative molecules.

Besides postsynaptic modulation, presynaptic 5-HT receptors (mainly 5-HT_1_ and 5-HT_2_ receptors) are involved in modulating the subsequent release of 5-HT and other neurotransmitters like glutamate and GABA appropriately in a feedback way. For example, presynaptic 5-HT_1A_ autoreceptors decreased neuronal firing and 5-HT release by opening G-protein-gated inwardly rectifying potassium channels (GIRKs) in rat dorsal raphe 5-HT neurons (Montalbano et al., 2015). 5-HT_1B_ autoreceptors were also found to inhibit AC and/or close Ca^2+^ channels to decrease presynaptic 5-HT release directly (Sharp et al., 2007). In addition, 5-HT could reduce the frequency of GABAergic mIPSCs and glutamatergic mEPSCs through 5-HT_1A_ and 5-HT_1B_ receptors, respectively, in a PKA-dependent pathway at the presynaptic terminals of the rat hypothalamus (Lee et al., 2008). Presynaptic 5-HT_2A_ receptor activation enhanced presynaptic NMDA receptor transmission and gated synaptic plasticity at thalamocortical synapses in the PFC (Barre et al., 2016). Besides this direct presynaptic modulation, 5-HT receptors coming from downstream non-5-HT neurons can affect the activity of upstream 5-HT neurons by the feedback projections indirectly (De Deurwaerdere and Di Giovanni, 2021). Taken together, 5-HT with its receptors have the great potential to modulate chronic pain and persistent anxiety bidirectionally by affecting presynaptic and postsynaptic transmission directly or indirectly. More efforts are needed to explore further neurobiological mechanisms.

### Effects of the 5-HT system on long-term potentiation and long-term depression

Synaptic plasticity, such as LTP and long-term depression (LTD), has been recognized as a key neural basis for sensory processing under physiological and pathological conditions (Zhuo, 2007). Chronic pain is always accompanied by the occurrence of CNS sensitization, such as the LTP in the ACC (Bliss et al., 2016). Hence, understanding the roles of the 5-HT system in the modulation of synaptic plasticity is also important for the treatment of chronic pain and its related emotion disorders. As a crucial neuromodulator, 5-HT displays great modulatory potentials in local synaptic plasticity (Barnes et al., 2021).

Currently, there are not so many studies about the serotonergic effects on the regulation of LTP, however, some studies in the spinal cord have revealed potential connections between 5-HT and LTP. For example, it was reported that low dose 5-HT could activate previously silent glutamatergic synapses and induce LTP by enhancing postsynaptic AMPA receptor function in the DH (Li and Zhuo, 1998; Li et al., 1999). At the pure NMDA synapses on sensory neurons in the spine, 5-HT alone does not produce any LTP, but co-application of 5-HT and AC activator forskolin produced long-lasting enhancement, including the recruitment of functional AMPA receptors. This recruitment emphasized the significance of the cAMP signaling pathway in 5-HT-mediated synaptic responses and raised a synergistic mechanism of LTP regulation (Wang and Zhuo, 2002). In the supraspinal regions, the 5-HT system is also indicated in the modulation of LTP, for example, Huang and Kandel (2007) found that 5-HT_4_ receptors mediated a late-phase LTP (L-LTP) in the mouse amygdala. Recently, Zhou et al. (2022) found that the increased 5-HT levels in mice ACC could restore LTP induction in the complete Freund’s adjuvant (CFA)-induced inflammatory pain model, which contributed to both pain and anxiety relief.

In addition, the 5-HT system has potential critical effects on the modulation of LTD. Tian et al. (2017) previously reported that bath application of 5-HT could produce inhibitory modulation at excitatory synapses in ACC. Both presynaptic and postsynaptic 5-HT receptors contributed to this inhibitory synaptic modulation (Tian et al., 2017). As a key form of synaptic plasticity, LTD is mainly classified into two types: one is NMDA receptor-mediated, and the other one is metabotropic glutamate receptor (mGluR)-mediated (Bliss et al., 2016). Barre et al. (2016) showed that stimulation of presynaptic 5-HT_2A_ receptors gated the induction of time-dependent LTD (t-LTD) mediated by NMDA receptors at thalamocortical synapses. At the excitatory synapses of PFC pyramidal neurons, 5-HT was found to induce LTD by cooperating with 5-HT_2_ receptors and mGluRs. This induction needed the p38 MAPK/Rab5-mediated enhancement of AMPA receptor internalization (Zhong et al., 2008).

Considering the complexity of 5-HT receptors and coupled signaling pathways, the exploration of 5-HT-mediated synaptic plasticity is still in its initial stages. More efforts are needed to further interpret serotonergic roles in the regulatory mechanism of synaptic plasticity, especially in the pain/anxiety-related cortical regions, such as the ACC, IC, and amygdala.

## Conclusion

In summary, recent studies consistently demonstrate that the 5-HT system has important effects on the multiple modulations of pain perception and injury-related anxiety. The serotonergic projections are often overlapped with pain-/anxiety-related pathways in the CNS, indicating both a strong correlation and functional overlap between them. The complexity of 5-HT receptors further provides a basis for diverse modulations of neural excitability and synaptic transmission. Among many causes leading to chronic pain and its related persistent anxiety, long-term sensitization in the peripheral and central nervous systems is still the crucial cause of persistent pain and mental disorders. In our opinion, NMDA receptor-mediated postsynaptic LTP and kainate receptor-mediated presynaptic LTP in the ACC are important for chronic pain perception and injury-related anxiety, respectively. It’s unclear whether these mechanisms of synaptic regulation are also taking place in other pain- or anxiety-related regions. More studies are needed to investigate the modulatory mechanisms of the 5-HT system at both the synaptic and molecular levels.

## Author contributions

SH, WS, WL, Q-YC, and MZ drafted the manuscript and finished the final version of the manuscript. All authors read and approved the final manuscript.
